# Supplementary material for: Global hypo-methylation in a proportion of glioblastoma enriched for an astrocytic signature is associated with increased invasion and altered immune landscape
Source: eLife. 2022 Nov 22;11:e77335. doi: 10.7554/eLife.77335 (PMC9681209; doi:10.7554/eLife.77335)
Supplement: Figure 2—source data 1. [file elife-77335-fig2-data1.zip › Figure_2_source_data_1/Figure_2A/homerResults/motif52.info.html]

Motif 52

## Information for 6-TGTGTGTGTTYTTGAGATRA (Motif 52)

C
A
G
T
C
A
T
G
A
C
G
T
A
C
T
G
A
C
G
T
A
C
T
G
A
C
G
T
A
C
T
G
A
C
G
T
A
C
G
T
A
G
T
C
A
G
C
T
G
C
A
T
A
C
T
G
C
T
G
A
C
T
A
G
T
C
G
A
A
C
G
T
C
T
A
G
C
G
T
A
  
Reverse Opposite:
